# Prevalence and determinants of anaemia among university students: a gender-based analysis

**DOI:** 10.3389/fpubh.2026.1780083

**Published:** 2026-03-11

**Authors:** Carine Platat, Ayah A. Ghanayem, Bakhita H. S. Alameri, Amal H. S. Alderei, Fatima G. S. Alderei, Alia M. A. Alkaabi, Meznah M. D. Almansoori, Rubina Sabir, Ihab Tewfik

**Affiliations:** 1 Department of Nutrition and Health, College of Medicine and Health Sciences, United Arab Emirates University, Al Ain, United Arab Emirates; 2 School of Life Sciences, University of Westminster, London, United Kingdom

**Keywords:** anaemia, body composition, dietary intake, gender, university students

## Abstract

**Introduction:**

Anaemia remains a major global public health concern, disproportionately affecting young women of reproductive age. University students represent a nutritionally vulnerable group; however, males are often underrepresented in anaemia surveillance. This study aimed to estimate the prevalence of anaemia among university students in the UAE and to identify gender-specific determinants.

**Methods:**

A cross-sectional study was conducted among students aged 18–35 years at a large university in the UAE. Sociodemographic data were collected via questionnaire. Haemoglobin concentration was measured using the HemoCue Hb 201 + system. Anthropometric measures and body composition were assessed, and dietary intake was evaluated using a 24-h recall.

**Results:**

The median age was 20.0 years (IQR: 19.0–22.0) and median BMI was 23.7 kg/m^2^ (IQR: 20.7–27.6). Compared with males, females were more likely to be underweight and less likely to be obese, exhibited lower fat-free, muscle, and bone mass, higher fat mass percentage, and lower dietary intakes. Overall anaemia prevalence was 49.10% (95%CI 45.30–52.90), with significantly higher rates among females (60.70, 95%CI 56.20–65.20) than males (24.60, 95%CI 18.80–30.40). No determinants were identified among females. In males, anaemia was inversely associated with height, fat-free mass, muscle mass, bone mass, and dietary iron and zinc intakes; height emerged as the strongest independent predictor.

**Conclusion:**

Anaemia is highly prevalent among UAE university students. These findings highlight the importance of including male students in screening and prevention strategies and adopting gender-specific approaches to anaemia control.

## 1 Introduction

Anaemia, characterised by low red blood cell count or haemoglobin levels, is a global public health issue with significant health, social, and economic consequences (1–3). The most common type is Iron Deficiency Anaemia (IDA), resulting from insufficient iron stores in the body (1). IDA accounts for nearly two-thirds of all anaemia cases worldwide, corresponding to around 1.3 billion people, i.e., about 16–17% of the global population (4). The World Health Organisation (WHO) defines anaemia based on haemoglobin levels: less than 12.0 g/dL for females and less than 13.0 g/dL for males (1). While anaemia can be mild and asymptomatic, it can cause fatigue, reduced physical capacity, and, in severe cases, shortness of breath and restless legs. Anaemia is also a risk factor for complications, mainly when it reflects other chronic conditions like cardiovascular disease or diabetes. In 2022, a study conducted in the UAE found that nearly half of cardiovascular patients (*n* = 104) were anaemic, and anaemia was a significant risk factor for complications such as chronic kidney failure and increased mortality (5).

The impact of anaemia varies by population. In older adults, it can exacerbate comorbidities, while pregnant women with anaemia may face premature labour and low birth weight in babies (6). Anaemia during pregnancy also increases the risk of anaemia in the baby. Chronic anaemia primarily affects the cardiovascular system, leading to complications like myocardial infarction and high-output heart failure. In severe cases, especially when present from a young age, anaemia can impair neurological development, leading to cognitive and developmental delays (2, 3, 61).

Anaemia affects 24.3% of the global population, with approximately 1.76 billion cases worldwide. Mild anaemia accounts for 954.3 million cases, moderate anaemia for 747.8 million, and severe anaemia for 59.5 million. Children under five and females are disproportionately affected, with prevalence rates of 31.2% in females and 17.5% in males. Developing regions such as Sub-Saharan Africa and South Asia bear the highest burden with an estimated 106 million women and 103 million children in Africa and 244 million women and 83 million children in South-East Asia affected by anaemia. These regions face challenges like inadequate nutrition, poor healthcare access, and a higher prevalence of infectious diseases (1).

In the Middle East, anaemia, particularly IDA, remains a significant health concern, especially among women of reproductive age, pregnant women, and children (1, 7). In 2021, the MENA region had 88.9 million cases of IDA, with the number of IDA cases in the UAE increasing from 359,399 in 1990 to 1.13 million in 2020 (more than two-fold in three decades), with females being more affected (7).

However, data on anaemia in the UAE remain limited. Available studies focus mainly on children and pregnant women. Reported prevalence range between 3 to 31% in children (8–10) and from 18.1 to 26.7% in female university students (11, 12). An hospital-based study reported a 31% prevalence of iron deficiency anaemia among hospitalised cases (13).

Although anaemia is commonly studied in women, its occurrence in males remains underexplored (14–16). The prevalence of anaemia is generally lower in men compared to women, but it still affects a significant and clinically relevant proportion of the male population. It affects 10.8% of Black non-Hispanic American males (17) and has recently been recognised as an emerging public health challenge in India, where it has reached 25% among men (18). In the UAE, one study reported a prevalence of 2.7% among Emirati adolescent males (19). Higher rates have been documented in a neighbouring country, with 44.9% among male adolescents and 11.1% among male university students in Saudi Arabia (20, 21). Including male participants is therefore important for understanding anaemia in university populations and for developing equitable public-health interventions.

Anaemia is a multifactorial condition influenced by nutritional deficiencies, chronic diseases, infections, malabsorption, genetic disorders, and socio-demographic factors. Nutritional deficiencies, particularly iron, vitamin A, folate, and vitamin B12, impair erythropoiesis, while iron absorption can be reduced by dietary inhibitors such as phytates and polyphenols in tea and coffee and enhanced by vitamin C. Inherited haemoglobinopathies, including sickle cell disease and thalassaemia, are also important contributors, especially in the Middle East where high rates of consanguinity remain a prevalent cultural tradition (1, 22, 23).

University students represent an important yet understudied population in anaemia research. This life stage is characterised by increased independence in food choices, academic stress, and financial constraints that may negatively affect diet and physical activity. Studies in the MENA region and in the UAE show that university students frequently skip meals, consume ultra-processed foods and sugar-sweetened beverages, and drink tea or coffee with meals, behaviours that may reduce micronutrient intake and impair iron absorption (24–32). Consequently, the dietary intake of key micronutrients, particularly iron, folate, vitamin A and riboflavin, remains inadequate across all age groups in the region (27, 33–36). Sedentary behaviour and reduced physical activity are also common, increasing the risk of weight gain and metabolic disturbances (28, 30). In female students, menstrual blood loss is another major physiological pathway leading to iron depletion (1).

Due to the multicultural context of the UAE, dietary habits are remarkably diverse. Different cultural groups often exhibit distinct dietary patterns, food preferences, and nutrient intakes, all of which can influence iron status and susceptibility to anaemia. In this context, nationality can serve as a meaningful proxy for cultural background and dietary practises, key factors in anaemia risk. Previous studies in Gulf countries have reported variation in anaemia prevalence between national and expatriate populations (37–39). By analysing the relationship between nationality and anaemia prevalence, this study aims to provide valuable insights into how cultural factors may shape the risk of anaemia among university students.

Parallel to anaemia, obesity has become one of the most pressing health challenges globally, particularly in the UAE. Over the past three decades, global obesity rates have nearly tripled, with an estimated 879 million adults classified as obese in 2022. If current trends continue, the number of adults living with overweight and obesity will reach 3.80 billion by 2050. The MENA region is among the most affected, with the UAE standing out with one of the highest adult obesity rates at 27.4% (40). Obesity-related inflammation may impair iron metabolism through increased hepcidin levels, linking excess body fat with iron-deficiency anaemia (41, 42). University students are prone to weight gain during their studies, a phenomenon documented across countries (43). The coexistence of excess weight with micronutrient deficiencies, the “double burden of malnutrition,” is increasingly recognised, as obesity-related inflammation alters iron metabolism and may contribute to anaemia. Given the frequent dietary imbalance among young adults in the UAE, examining body composition together with dietary intake is particularly relevant when studying anaemia in university populations (11).

This study aims to investigate the prevalence of anaemia among university students in the United Arab Emirates while examining the gender-specific relationships with dietary intake, body composition, and nationality. By focusing on university students, this study addresses an important evidence gap and explores behavioural and biological determinants of anaemia among young adults. Crucial in developing targeted public health interventions.

## 2 Materials and methods

### 2.1 Study design and participants

A cross-sectional study was conducted among male and female students at the United Arab Emirates University (UAEU) in Al Ain during the Spring and Fall of 2024. A convenience sample of university students was recruited between August 26th and November 20th, 2024. Flyers were displayed across campus, including near elevators and in dining areas, to advertise the study. Recruitment was further supported by trained research assistants who approached students and invited them to participate. Participants were eligible if they were registered at UAEU, aged between 18 and 35 years, and met the following criteria: No diagnosed eating disorders; no known red blood cell disorders (such as *α*- or *β*-thalassaemia or sickle cell disease); no diagnosis of hemochromatosis; no severe or recent infection within the past 6 months; not currently pregnant or breastfeeding (for females); no regular use of medications or nutritional supplements; and no blood donation within the past 6 months.

The study was approved by the Social Sciences Research Ethics Committee (ERSC_2024_4315) and the Human Research Ethics Committee (ERH_2024_4314) at UAEU. All participants received detailed information about the study and related procedures, both orally and in writing, and were allowed to ask questions to the research team. Written informed consent was obtained from each participant before data collection. Data collection was conducted either immediately after consent on the day of recruitment or at a scheduled time convenient for the participant.

**Figure 1:**
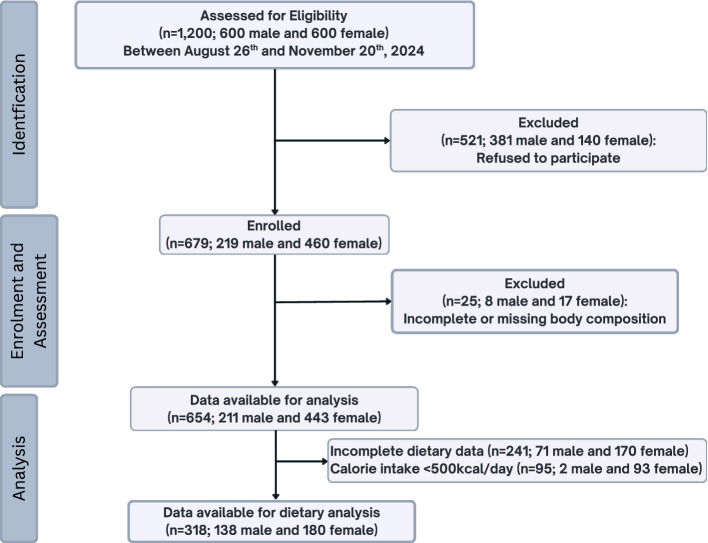
Study flowchart.

A total of 1,200 participants (equal numbers of males and females) were screened for eligibility (Figure 1). Of these, 381 males and 140 females declined to participate. Consequently, 679 university students, including 219 males and 460 females, were initially recruited for the study. After excluding participants (8 males and 17 females) with incomplete or missing body composition data, the final sample size comprised 654 participants (211 males and 443 females). The sample size was deemed sufficient to estimate the prevalence of anaemia in both genders, which is the primary objective of this study, with acceptable precision. For males, an expected anaemia prevalence of 11.1%, based on previous regional studies (21), and a sample size of 211 allowed estimation with a 95% confidence interval and a precision of ±4.2%. For females, an expected prevalence of 26.7% (12) yielded an accuracy of ±4.1% with 443 participants. These margins of error are considered statistically acceptable for descriptive cross-sectional studies. Additionally, the final sample size provided adequate power to detect gender differences in anaemia prevalence using appropriate statistical comparisons. Complete dietary data were available for 318 participants (138 males and 180 females). Analyses examining the relationship between dietary intake and anaemia were conducted within this sub-sample, after confirming that there were minimal differences between the sub-sample and the remaining participants in the study (*n* = 336). Details are provided in the statistical analysis section.

### 2.2 Demographic data

The research team collected data on a single occasion. An online questionnaire was used to collect basic demographic information, including age, gender, and nationality.

### 2.3 Anthropometry

Height was measured using a portable SECA Stadiometer (SECA 213, SECA GmbH & Co. KG, Hamburg, Germany). Weight and body composition were assessed using the bioelectrical impedance method with a Tanita Body Composition Analyser (MC-780MA, Tanita Corp., Tokyo, Japan).

### 2.4 Anaemia status

Haemoglobin (Hb) levels were measured using the HemoCue Hb 201 + System Analyser (HemoCue AB, Ängelholm, Sweden), which determines Hb concentration from capillary blood obtained by finger-prick sampling. This method was selected because it is practical, convenient, and cost-effective for large population studies and is widely used in epidemiological field settings. Blood collection follows a protocol similar to that used for capillary blood glucose testing. All research staff were trained in aseptic technique, safe capillary sampling, and device operation before data collection. Disposable single-use lancets and microcuvettes were used, and samples were analysed immediately after collection according to manufacturer instructions. Quality-control procedures included daily instrument calibration checks using control cuvettes and routine verification with manufacturer-provided control solutions. Any values outside the acceptable range were rechecked using a second capillary sample. Participants were instructed to refrain from vigorous physical activity for at least 24 h before the assessment to minimise acute haemoconcentration effects on Hb measurements.

Anaemia was defined using WHO haemoglobin thresholds (44): Hb < 12.0 g/dL for females and Hb < 13.0 g/dL for males. Anaemia severity was additionally classified according to WHO criteria as mild, moderate, or severe anaemia. For females, mild anaemia was defined as Hb 11.0–11.9 g/dL, moderate as 8.0–10.9 g/dL, and severe as <8.0 g/dL. For males, mild anaemia was defined as Hb 11.0–12.9 g/dL, moderate as 8.0–10.9 g/dL, and severe as <8.0 g/dL.

For regression analyses, anaemia was coded as a binary variable (anaemia vs. no anaemia), while severity categories were used for descriptive analyses only.

### 2.5 Dietary intake

A single 24-h dietary recall was administered. Trained undergraduate students from the Department of Nutrition and Health at UAEU served as interviewers after completing formal training in dietary assessment techniques. Training covered the multiple-pass recall method, portion-size estimation, and probing for commonly forgotten foods. Standardised instructions and practise sessions were conducted before data collection to ensure consistency across interviewers.

Nutrient intakes were analysed using ESHA Food Processor software (ESHA Research, Salem, OR, United States). The intake of macronutrients and selected micronutrients (vitamins A, Riboflavin, B12, C, D, E, folate and minerals including iron, zinc, and copper) was calculated. The Calorie and macronutrient intake were expressed in kcal and g, respectively, and in kcal and g per kg of body weight (BW). Dietary adequacy was assessed using Dietary Reference Intakes (DRIs) relevant to each nutrient (45). Quality-control procedures included double data entry for a random subsample and independent verification of nutrient calculations. Under- and over-reporting were screened using energy intake relative to estimated energy requirements, and extreme values were checked before analysis.

### 2.6 Statistical analysis

Data were analysed using IBM SPSS Statistics for Windows, version 26.0 (IBM Corp., Armonk, NY, United States). Results are presented as mean ± standard deviation (SD), median (interquartile range, IQR), or percentages, as appropriate. Categorical variables are presented as prevalence with 95% confidence intervals. Data normality was assessed using the Shapiro–Wilk test, and all continuous variables were found to be non-normally distributed.

Anaemia prevalence was reported as the proportion of males and females with Hb levels below the WHO-defined thresholds. Associations between anaemia status and variables, including age, gender, and body composition, were assessed using the non-parametric Mann–Whitney U test. The relationship between anaemia and nationality was evaluated using the Chi-Square test.

Dietary intake was assessed in a sub-sample of 318 participants (138 males and 180 females) (Figure 1). This sub-sample was not selected *a priori* but resulted from missing responses during data collection or the exclusion of participants who reported a calorie intake of less than 500 kcal/day or above 5,000 kcal/day. To assess potential selection bias, the sub-sample was compared with the remainder of the study sample (*n* = 336) in terms of age, nationality, BW, and anaemia prevalence for each gender. No significant differences were found in age, height, fat mass percentage, fat-free mass, muscle mass, bone mass, Hb level and prevalence of anaemia. Only the proportion of Emirati females was lower in the sub-sample compared to the remainder of the sample. Representation of Emirati nationality (60.60% versus 78.70% among the remainders, *p* < 10^−3^).

Associations between anaemia and dietary intake were also analysed using the Mann–Whitney U test. Binary logistic regression models were constructed to identify independent predictors of anaemia in the full sample and sub-group. Variables with *p* < 0.05 in univariate analyses were entered into multivariable models. Multicollinearity was assessed using variance inflation factors (VIF), with VIF > 5 indicating potential collinearity. Model goodness of fit was evaluated using the Hosmer–Lemeshow test, and model discrimination was assessed using the area under the receiver operating characteristic curve (AUC/ROC). Adjusted odds ratios (ORs) with 95% confidence intervals (CIs) were reported.

## 3 Results

A total of 679 students were recruited for the study. The sample size was deemed sufficient to estimate the prevalence of anaemia in both genders with acceptable precision. For males, an expected anaemia prevalence of 11.1%, based on previous regional studies (24), and a sample size of 211 allowed estimation with a 95% confidence interval and a precision of ±4.2%. For females, an expected prevalence of 26.7% (20) yielded an accuracy of ±4.1% with 443 participants. These margins of error are considered statistically acceptable for descriptive cross-sectional studies. Additionally, the final sample size provided adequate power to detect gender differences in anaemia prevalence using appropriate statistical comparisons.

The study population included 654 university students, comprising 211 males and 443 females, with the majority being Emirati nationals (67.0%) (Table 1). The median Hb level for the entire sample was 12.10 g/dL (IQR: 11.10–13.50). As expected, Hb levels were significantly higher in males (14.10 g/dL (IQR: 13.00–15.00)) than in females (11.70 g/dL (IQR: 10.80–12.50)) (*p* < 0.001). The overall prevalence of anaemia was 49.10% (95%CI 45.30–52.90) among 654 participants. Anaemia prevalence was 24.60% (95% CI 18.80–30.40) in males and 60.70% (95% CI 56.20–65.20) in females, with a significantly higher prevalence in females than males (*p* < 0.05).

**Table 1:** Characteristics of the study population.

Parameter	Total (*n* = 654)	Male (*n* = 211)	Female (*n* = 443)
Age (y)	20.00 (19.00–22.00)	21.00 (19.00–22.00)	20.00 (19.00–21.00)
Emirati (%)	67.00	55.90	72.21^a^
Hb (g/dL)	12.10 (11.10–13.50)	14.10 (13.00–15.00)	11.70 (10.80–12.50)^a^
Anaemia (%)	49.10 (95%CI 45.30–52.90)	24.60 (95%CI 18.80–30.40)	60.70 (95%CI 56.20–65.20)^a^
Mild	29.40 (95%CI 25.90–32.90)	21.30 (95%CI 15.80–26.80)	33.20 (95%CI 28.80–37.60)^a^
Moderate	17.70 (95%CI 14.80–20.60)	1.90 (95%CI 0.10–3.80)	25.30 (95%CI 21.30–29.30)
Severe	2.00 (95%CI 0.90–3.10)	1.40 (95%CI 0.00–3.00)	2.30 (95%CI 0.90–3.60)
Height (cm)	161.00 (156.40–167.83)	171.10 (166.30–177.40)	158.30 (154.90–161.60)^a^
Weight (kg)	62.00 (52.38–74.93)	74.20 (64.90–87.90)	57.50 (49.90–67.60)^a^
BMI (kg/m^2^)	23.70 (20.70–27.63)	25.30 (22.60–28.50)	22.80 (20.20–27.10)
Underweight (%)	9.90	5.70	12.00^a^
Normal weight (%)	48.90	39.30	53.50
Overweight (%)	25.50	36.00	20.50
Obese (%)	15.60	19.00	14.00
Fat mass (%)	23.60	19.05	26.30^a^
Fat mass (kg)	15.00 (10.00–21.75)	14.50 (10.00–21.10)	15.30 (10.00–22.60)
Fat-free mass (kg)	44.40 (40.38–53.60)	59.60 (52.90–66.50)	41.90 (39.20–45.10)^a^
Muscle mass (kg)	42.40 (38.80–51.63)	56.80 (50.50–63.40)	40.00 (37.60–42.90)^a^
Bone mass (kg)	2.30 (2.10–2.70)	3.00 (2.70–3.30)	2.20 (2.00–2.30)^a^

Data are presented as median (interquartile range, IQR), anaemia prevalence as % with 95% confidence intervals (95%CI) or percentages (%), as appropriate. * Comparison between genders was made using the Mann–Whitney U test.

a Significant statistical difference between genders, *p* < 0.05.

The median height was 161.00 cm (IQR: 156.40–167.83) in the total sample, with males being significantly taller (171.10 cm (IQR: 166.30–177.40)) than females (158.30 cm (IQR: 154.90–161.60)) (*p* < 0.001). Regarding nutritional status, nearly half of the population had a normal BMI, while 9.60% were classified as underweight, 24.00% as overweight, and 15.60% as obese. A greater proportion of females had a normal BMI compared to males. Conversely, overweight and obesity, particularly overweight, were more prevalent among male participants. Underweight was more common among females.

In terms of body composition, the median fat mass in kg was 15.00 kg (IQR: 10.00–21.75). No difference was reported between females and males; however, the fat mass percentage was greater in females, at 26.30% (IQR: 20.00–33.30), compared to males, at 19.05% (IQR: 14.30–24.15) (*p* < 0.001), likely due to the significantly lower weight among females versus males. Median fat-free mass, muscle mass and bone mass were 44.40 kg (IQR: 40.38–53.60), 42.40 kg (IQR: 38.80–51.63) and 2.30 kg (2.10–2.70), respectively, with lower values in females compared to males.

Nationality was not significantly related to anaemia status (*p* = 0.80), neither in males nor in females (*p* = 0.34 and *p* = 0.59, respectively) (data not shown).

Among females, no significant associations were observed between anaemia status and age, height, or body composition parameters (Table 2). In contrast, several body composition measures were significantly related to anaemia in males (Table 3). Specifically, lower height (*p* < 10^−3^), fat-free mass (*p* < 10^−2^), muscle mass (*p* < 10^−2^), and bone mass (*p* < 10^−2^) were associated with the presence of anaemia.

**Table 2:** Relationship between anaemia, age and body composition in females (*n* = 443)*.

Parameter	Anaemia	*N*	Median (IQR)	*Z*	*r*	*p*-value
Age (y)	No anaemia	174	20.00 (19.00–21.00)	−0.20	−0.01	0.84
	Anaemia	269	20.00 (19.00–21.00)			
Height (cm)	No anaemia	174	158.20 (155.15–162.15)	−0.26	−0.01	0.80
	Anaemia	269	158.35 (154.90–161.50)			
Weight (kg)	No anaemia	174	57.60 (49.30–67.40)	−0.28	−0.01	0.78
	Anaemia	269	57.15 (50.10–67.73)			
BMI (kg/m^2^)	No anaemia	174	22.50 (19.85–26.90)	−0.61	−0.03	0.54
	Anaemia	269	22.80 (20.43–27.10)			
Fat mass (%)	No anaemia	174	27.40 (19.50–33.50)	−0.54	−0.03	0.59
	Anaemia	269	25.45 (20.03–33.20)			
Fat mass (kg)	No anaemia	174	15.90 (10.00–23.25)	−0.43	−0.02	0.67
	Anaemia	269	14.15 (10.10–21.98)			
Fat-free mass (kg)	No anaemia	174	41.60 (38.60–44.40)	−1.42	−0.07	0.16
	Anaemia	269	42.10 (39.53–45.18)			
Muscle mass (kg)	No anaemia	174	39.90 (37.05–42.35)	−1.70	−0.08	0.09
	Anaemia	269	40.35 (38.10–43.00)			
Bone Mass (kg)	No anaemia	174	2.10 (2.00–2.30)	−1.84	−0.09	0.07
	Anaemia	269	2.20 (2.03–2.30)			

* Comparison between anaemia status was made using the Mann–Whitney U test, *Z* = *Z*-score; *r* = Effect size; IQR = Interquartile Range.

**Table 3:** Relationship between anaemia, age and body composition in males (*n* = 211)*.

Parameter	Anaemia	*N*	Median (IQR)	*Z*	*r*	*p*-value
Age (y)	No anaemia	159	21.00 (19.00–22.00)	−1.11	−0.08	0.27
	Anaemia	52	21.00 (19.00–23.00)			
Height (cm)	No anaemia	159	172.40 (167.00–178.00)	−3.68	−0.25	<10^−3^
	Anaemia	52	168.20 (161.58–174.33)			
Weight (kg)	No anaemia	159	74.20 (66.70–88.15)	−1.24	−0.09	0.21
	Anaemia	52	73.80 (61.08–81.30)			
BMI (kg/m^2^)	No anaemia	159	25.20 (22.45–28.45)	−0.29	−0.02	0.77
	Anaemia	52	25.30 (22.53–28.23)			
Fat mass (%)	No anaemia	159	18.80 (14.15–23.30)	−1.15	−0.08	0.25
	Anaemia	52	21.40 (14.45–28.23)			
Fat mass (kg)	No anaemia	159	14.20 (9.95–20.50)	−0.03	0.00	0.98
	Anaemia	52	15.90 (8.55–21.15)			
Fat-free mass (kg)	No anaemia	159	60.00 (53.95–67.40)	−2.96	−0.20	<10^−2^
	Anaemia	52	57.05 (46.78–63.10)			
Muscle mass (kg)	No anaemia	159	57.10 (51.95–64.30)	−3.10	−0.21	<10^−2^
	Anaemia	52	54.15 (45.08–59.98)			
Bone mass (kg)	No anaemia	159	3.00 (2.75–3.35)	−3.10	−0.21	<10^−2^
	Anaemia	52	2.90 (2.40–3.13)			

* Comparison between anaemia status was made using the Mann–Whitney U test. *Z* = Z-score; *r* = Effect size; IQR = Interquartile Range.

A sub-group of 318 participants was considered for the analysis of dietary data. The median total caloric intake was 1634.05 kcal (IQR: 1291.20–2189.00), with higher values observed in males compared to females (Table 4). However, when adjusted for total BW, energy intake was comparable between the two genders. Median intakes of carbohydrates, fat, and protein in grams exceeded the recommended values for both males and females.

**Table 4:** Dietary intake of the sub-group of participants (*n* = 318).

Parameter	Total (*n* = 318)	Male *n* = 138	Female *n* = 180	DRI (34)
CAL^†^ (kcal)	1634.05 (1291.20–2189.00)	1925.07 (1456.79–2702.09)	1497.45 (1194.24–1892.47)^a^	–
CAL^†^ (kcal/kgBW)	26.62 (18.43–35.77)	27.80 (17.54–36.33)	26.27 (18.54–35.35)	–
CHO^‡^ (g)	188.54 (129.74–262.51)	223.45 (139.84–297.94)	176.00 (122.40–231.33)^a^	130
CHO^‡^ (g/kgBW^¶^)	2.99 (1.94–4.19)	3.02 (1.71–4.38)	2.97 (2.01–4.17)	–
CHO^‡^ (% kcal)	46.32 (30.63–52.98)	45.32 (37.99–53.55)	47.44 (41.45–52.94)	45–65
PROT^§^ (g)	62.67 (43.06–92.00)	85.19 (51.91–115.33)	54.01 (38.15–76.84)^a^	56 (M**) 46 (F***)
PROT^§^ (g/kgBW^¶^)	1.01 (0.67–1.48)	1.12 (0.75–1.55)	0.87 (0.60–1.40)^a^	–
PROT^§^ (% kcal)	15.44 (11.95–20.29)	17.64 (13.34–21.83)	14.27 (11.34–18.53)^a^	10-35
Fat (g)	63.22 (43.38–88.13)	72.26 (54.56–100.72)	55.69 (39.10–78.90)^a^	–
Fat (g/kg BW^¶^)	0.97 (0.63–1.47)	1.01 (0.61–1.41)	0.95 (0.65–1.50)	–
Fat (% kcal)	15.70 (13.02–18.22)	15.25 (12.45–18.53)	15.91 (13.26–17.95)	20–35
Water (L)	1.50 (0.75–2.00)	2.00 (1.50–2.50)	1.00 (0.53–1.50)^a^	3.7 (M) 2.7 (F)
Vit.^**|**^ A (mcg)	104.88 (31.30–291.27)	126.33 (39.38–392.71)	98.59 (30.09–219.75)^a^	900 (M) 700 (F)
Riboflavin (mg)	0.66 (0.29–1.10)	0.84 (0.41–1.30)	0.55 (0.24–0.91)^a^	1.3 (M) 1.1 (F)
Vit.^**|**^ B12 (mg)	0.97 (0.19–2.40)	1.31 (0.32–3.32)	0.62 (0.09–1.96)^a^	2.4
Folate (mcg)	130.55 (31.51–262.18)	177.80 (44.02–360.99)	111.50 (23.30–203.58)^a^	400
Vit.^**|**^ D (mcg)	0.49 (0.00–2.55)	0.71 (0.00–3.31)	0.29 (0.00–1.93)^a^	15
Vit.^**|**^ E (mg)	1.97 (0.67–4.34)	2.50 (1.00–5.69)	1.78 (0.45–3.33)^a^	15
Vit.^**|**^ C (mg)	19.56 (4.83–75.36)	27.63 (5.88–84.00)	14.68 (4.73–69.82)^a^	90 (M) 75 (F)
Iron (mg)	7.89 (4.94–12.34)	9.59 (5.67–16.55)	7.08 (4.08–10.00)^a^	8 (M) 18 (F)
Zinc (mcg)	3.16 (1.51–6.20)	5.12 (2.29–7.99)	2.54 (1.36–4.60)^a^	11 (M) 8 (F)
Copper (mg)	0.45 (0.23–0.75)	0.58 (0.34–1.02)	0.35 (0.18–0.61)^a^	0.90

Data are presented as median (interquartile range, IQR).

^†^ CAL: calories; ^‡^ CHO: Carbohydrates; ^§^PROT: Protein; ^**|**^Vit.: Vitamin; ^¶^BW: Body Weight; **M: Male; ***F: Female.

* Comparison between genders was made using the Mann–Whitney U test.

a Significant statistical difference between genders, *p* < 0.05.

When expressed as a percentage of total caloric intake, carbohydrate contribution fell within the recommended range and tended toward the lower end for both sexes. Carbohydrate and fat intake in absolute grams, but not when expressed per kilogramme of BW, were significantly higher in males (72.26 g (IQR: 54.56–100.72)) than in females (55.69 g (IQR: 39.10–78.90)).

The fat contribution to total energy intake was below the recommended range for all participants (median 15.70% (IQR: 13.02–18.22)), with no significant gender differences.

Protein intake in grams was above recommended levels for both males (85.19 g (IQR: 51.91–115.33)) and females (54.01 g (IQR: 38.15–76.84), *p* < 0.05). However, when expressed as a percentage of total energy intake, protein remained within the recommended range for both genders, though significantly higher in males (17.64% (IQR: 13.34–21.83)) than in females (14.27% (IQR: 11.34–18.53), *p* < 0.05).

Median water intake was well below the recommended daily amounts of 3.7 L for males and 2.7 L for females.

Except for iron intake in males, all assessed micronutrients were consumed in insufficient amounts by both sexes. Micronutrient intakes were consistently lower among females compared to males.

Except for dietary intake of iron and zinc in males, no significant associations were observed between anaemia and dietary intake in either gender (Tables 5, 6). Specifically, iron and zinc intakes were significantly lower among males with anaemia compared to those without (*p* = 0.03 for dietary iron intake and *p* = 0.04 for dietary zinc intake).

**Table 5:** Relationship between anaemia and dietary intake in females (*n* = 180)*.

Parameter	Anaemia	*N*	Median (IQR)	*Z*	*r*	*p*-value
CAL^†^ (kcal)	No anaemia	72	1529.00 (1216.07–1882.79)	−0.24	−0.02	0.81
	Anaemia	108	1485.81 (1165.00–1884.16)			
CAL^†^ (kcal/kg BW)	No anaemia	72	25.53 (19.62–33.37)	−0.04	0.00	0.97
	Anaemia	108	24.98 (18.27–35.29)			
CHO^‡^ (g)	No anaemia	72	175.00 (120.54–228.14)	−0.15	−0.01	0.88
	Anaemia	108	175.64 (121.90–235.75)			
CHO^‡^ (g/kg BW)	No anaemia	72	2.95 (1.95–3.90)	−0.41	−0.03	0.68
	Anaemia	108	2.95 (2.03–4.20)			
CHO^‡^ (% kcal)	No anaemia	72	47.89 (41.87–52.95)	−0.15	−0.01	0.88
	Anaemia	108	47.41 (41.46–53.04)			
PROT^§^ (g)	No anaemia	72	54.00 (38.44–74.50)	−0.10	−0.01	0.92
	Anaemia	108	54.01 (36.32–78.81)			
PROT^§^ (g/kg BW)	No anaemia	72	0.90 (0.59–1.31)	−0.06	0.00	0.96
	Anaemia	108	0.87 (0.59–1.40)			
PROT^§^ (% kcal)	No anaemia	72	13.87 (11.34–19.33)	−0.32	−0.02	0.75
	Anaemia	108	14.67 (11.27–18.07)			
Fat (g)	No anaemia	72	55.96 (42.00–76.35)	−0.22	−0.02	0.83
	Anaemia	108	54.44 (38.06–79.90)			
Fat (g/kg BW)	No anaemia	72	0.95 (0.71–1.34)	−0.10	−0.01	0.92
	Anaemia	108	0.92 (0.59–1.59)			
Fat (% kcal)	No anaemia	72	35.06 (28.87–39.74)	−0.88	−0.07	0.38
	Anaemia	108	36.20 (29.87–41.50)			
Water intake (L)	No anaemia	72	1.00 (0.50–1.50)	−0.29	−0.02	0.77
	Anaemia	108	1.00 (0.58–1.50)			
Vit.^**|**^ A (mcg)	No anaemia	72	100.93 (31.49–205.11)	−0.54	−0.04	0.59
	Anaemia	108	93.50 (15.85–236.25)			
Riboflavin (mg)	No anaemia	72	0.53 (0.23–0.89)	−0.28	−0.02	0.78
	Anaemia	108	0.55 (0.23–0.91)			
Vit.^**|**^ B12 (mg)	No anaemia	72	0.68 (0.07–1.80)	−0.36	−0.03	0.72
	Anaemia	108	0.53 (0.09–2.02)			
Vit.^**|**^ C (mg)	No anaemia	72	20.30 (4.69–74.01)	−0.73	−0.05	0.46
	Anaemia	108	11.40 (4.68–65.00)			
Vit.^**|**^ D (mcg)	No anaemia	72	0.25 (0.00–2.10)	−0.28	−0.02	0.78
	Anaemia	108	0.29 (0.00–1.83)			
Vit.^**|**^ E (mg)	No anaemia	72	1.80 (0.38–3.47)	−0.11	−0.01	0.91
	Anaemia	108	1.84 (0.64–3.31)			
Folate (mcg)	No anaemia	72	97.25 (33.80–196.00)	−0.03	0.00	0.98
	Anaemia	108	119.50 (15.08–208.77)			
Iron (mg)	No anaemia	72	6.80 (4.08–10.00)	−0.08	−0.01	0.94
	Anaemia	108	7.08 (3.86–10.05)			
Zinc (mcg)	No anaemia	72	2.40 (0.93–4.15)	−0.47	−0.04	0.64
	Anaemia	108	2.54 (1.45–4.61)			
Copper (mcg)	No anaemia	72	0.38 (0.19–0.64)	−0.85	−0.06	0.39
	Anaemia	108	0.33 (0.18–0.59)			

† CAL: calories; ^‡^CHO: Carbohydrates; ^§^PROT: Protein; ^**|**^Vit.: Vitamin; ^¶^BW: Body Weight. *Comparison between anaemia status was made using the Mann–Whitney U test, *Z* = Z-score; *r* = Effect size; IQR = Interquartile Range.

**Table 6:** Relationship between anaemia and dietary intake in males (*n* = 138).

Parameter	Anaemia	*N*	Median (IQR)	*Z*	*r*	*p*-value
CAL^†^ (kcal)	No anaemia	106	1979.63 (1466.29–2711.22)	−1.09	−0.09	0.28
	Anaemia	32	1667.82 (1247.01–2298.62)			
CAL^†^ (kcal/kg BW)	No anaemia	106	28.61 (18.57–36.64)	−0.63	−0.05	0.53
	Anaemia	32	21.05 (15.27–33.80)			
CHO^‡^ (g)	No anaemia	106	222.76 (143.80–286.72)	−0.04	0.00	0.97
	Anaemia	32	210.23 (122.94–313.75)			
CHO^‡^ (g/kg BW)	No anaemia	106	2.94 (1.74–4.00)	−0.35	−0.03	0.72
	Anaemia	32	3.04 (1.35–4.54)			
CHO^‡^ (% kcal)	No anaemia	106	45.12 (37.31–52.86)	−1.13	−0.10	0.26
	Anaemia	32	45.30 (38.44–55.00)			
PROT^§^ (g)	No anaemia	106	86.06 (57.24–115.61)	−1.38	−0.12	0.17
	Anaemia	32	66.30 (41.13–104.89)			
PROT^§^ (g/kg BW)	No anaemia	106	1.12 (0.80–1.55)	−0.93	−0.08	0.36
	Anaemia	32	1.06 (0.49–1.25)			
PROT^§^ (% kcal)	No anaemia	106	17.91 (13.36–21.79)	−0.46	−0.04	0.65
	Anaemia	32	16.26 (13.18–24.95)			
Fat (g)	No anaemia	106	71.58 (55.03–103.79)	−0.72	−0.06	0.47
	Anaemia	32	66.80 (40.45–93.07)			
Fat (g/kg BW)	No anaemia	106	1.03 (0.68–1.42)	−0.54	−0.05	0.59
	Anaemia	32	0.85 (0.57–1.34)			
Fat (% kcal)	No anaemia	106	34.70 (27.91–42.10)	−0.31	−0.03	0.76
	Anaemia	32	33.83 (29.50–44.23)			
Water intake (L)	No anaemia	106	2.00 (1.50–2.50)	−0.86	−0.07	0.39
	Anaemia	32	2.00 (1.00–2.50)			
Vit.^**|**^ A (mcg)	No anaemia	106	135 (45.38–388.00)	−0.32	−0.03	0.75
	Anaemia	32	91.87 (4.79–436.12)			
Riboflavin (mg)	No anaemia	106	0.85 (0.41–1.30)	−1.19	−0.10	0.23
	Anaemia	32	0.68 (0.33–1.05)			
Vit.^**|**^ B12 (mg)	No anaemia	106	1.43 (0.45–3.42)	−1.38	−0.12	0.17
	Anaemia	32	0.52 (0.22–3.08)			
Vit.^**|**^ C (mg)	No anaemia	106	23.33 (5.50–84.80)	−0.43	−0.04	0.66
	Anaemia	32	32.86 (1.43–76.39)			
Vit.^**|**^ D (mcg)	No anaemia	106	0.75 (0.02–3.21)	−0.40	−0.03	0.69
	Anaemia	32	0.70 (0.00–3.61)			
Vit.^**|**^ E (mg)	No anaemia	106	2.35 (1.06–2.35)	−0.19	−0.02	0.85
	Anaemia	32	2.50 (0.48–9.72)			
Folate (mcg)	No anaemia	106	197.28 (49.79–348.68)	−0.60	−0.05	0.55
	Anaemia	32	135.90 (32.50–405.68)			
Iron (mg)	No anaemia	106	10.47 (6.49–16.71)	−2.22	−0.19	0.03
	Anaemia	32	5.92 (3.84–13.86)			
Zinc (mcg)	No anaemia	106	5.31 (2.95–8.00)	−2.04	−0.17	0.04
	Anaemia	32	2.38 (1.12–7.46)			
Copper (mcg)	No anaemia	106	0.58 (0.35–0.94)	−0.61	−0.05	0.54
	Anaemia	32	0.42 (0.22–1.21)			

^†^CAL: calories; ^‡^CHO: Carbohydrates; ^§^PROT: Protein; ^**|**^Vit.: Vitamin; ^¶^BW: Body Weight. * Comparison between anaemia status was made using the Mann–Whitney U test, Z = Z-score; *r* = Effect size, IQR = Interquartile Range.

Binary logistic regression models were constructed for males only, as no variables were significantly associated with anaemia in females, neither in the full sample nor in the subgroup (data not shown). Muscle mass and bone mass showed high collinearity (VIF = 11.7 and 15.0), indicating redundancy with fat free mass. Therefore, only fat free mass was retained in the models.

The first model (Table 7), developed in the full male sample, included height and fat-free mass. Only height (*p* ≤ 0.01) remained significantly associated with anaemia. Each 5-cm increase in height was associated with 34% lower odds of anaemia (OR = 0.66, 95% CI 0.50–0.89, *p* ≤ 0.01), while fat-free mass was not associated with anaemia (OR = 0.99, 95% CI 0.96–1.03, *p* = 0.57). The model showed good calibration (Hosmer–Lemeshow χ^2^ = 5.85, *p* = 0.66) and acceptable discrimination (AUC = 0.68, 95% CI 0.58–0.79, *p* = 0.001).

**Table 7:** Association between body composition and anaemia among male students (*n* = 211; subsample, *n* = 138): results from logistic regression.

Parameter	B	S.E.	OR^†^ (95%CI^‡^)	*p*-value
Height (cm)	−0.08	0.03	0.92 (0.87–0.98)	≤10^−2^
			OR per 5 cm=	
			0.66 (0.50–0.89)	
Fat-free mass (kg)	−0.001	0.02	0.99 (0.96–1.03)	0.57
Sub-sample				
Height (cm)	−0.09	0.04	0.91 (0.84–0.99)	0.02
			OR per 5 cm=	
			0.63 (0.42–0.93)	
Fat-free mass (kg)	−0.01	0.02	0.99 (0.95–1.04)	0.69
Iron (mg)	−0.05	0.04	0.96 (0.88–1.04)	0.29
Zinc (mg)	0.04	0.05	1.05 (0.94–1.16)	0.42

^†^OR, odd-ratio; ^‡^CI, confidence interval for the odds ratio.

A second model (Table 7) was developed using the male sub-sample for which dietary data were available, to examine the additional effects of dietary iron and zinc intake. After including these dietary variables, height (*p* = 0.02) remained the only factor significantly associated with anaemia. Each 5-cm increase in height was associated with 37% lower odds of anaemia (OR = 0.63, 95% CI 0.42–0.93, *p* = 0.02), while fat-free mass, iron intake, and zinc intake were not significantly associated with anaemia. This model also demonstrated adequate fit (Hosmer–Lemeshow χ^2^ = 9.43, *p* = 0.31) and similar discrimination (AUC = 0.69, 95% CI 0.58–0.79, *p* = 0.001).

## 4 Discussion

This study provides the first prevalence data on anaemia among a large sample of university students in the UAE. An alarming prevalence was observed, with half of the population having anaemia and with significantly higher rates in females (60.70, 95%CI 56.20–65.20) than in males (24.60, 95%CI 18.80–30.40). In males, several factors, including height, fat-free mass, muscle mass, bone mass, and dietary intake of iron and zinc, were inversely associated with anaemia. Further multivariable analysis revealed that height was the strongest independent predictor of anaemia among male students. These results underline the importance of not overlooking male students in screening and prevention strategies, as they may present distinct yet significant risk profiles. Moreover, the results highlight the critical role of body composition, particularly height, and nutritional factors, such as iron and zinc intake, in determining the risk of anaemia among males in university settings. Addressing these gender-specific determinants is essential for developing effective and inclusive public health strategies.

### 4.1 A high anaemia prevalence

Several factors may explain the high anaemia prevalence among university students at UAEU. First, haemoglobin was assessed using capillary blood, which is widely used in field studies (46–48), but can yield slightly lower or more variable Hb values than venous measurements due to differences in sampling technique, haemoconcentration, or peripheral circulation, potentially increasing anaemia estimates in population screening (49, 50). Second, characteristics of the university population may contribute to higher prevalence. University students frequently report irregular meal patterns, low intake of iron-rich foods, high consumption of tea or coffee with meals, dieting behaviours, sedentary lifestyles, and weight gain during their studies, all of which can reduce micronutrient intake or iron bioavailability (24–32). Third, biological factors are particularly relevant in this age group. Young women are vulnerable to iron deficiency due to menstrual blood loss (1). Obesity-related inflammation may impair iron metabolism through elevated hepcidin levels in a population with a large rate of obesity (41, 42). In a multicultural population such as the UAE, inherited haemoglobinopathies and recent infections may also contribute to anaemia risk. Together, these methodological, behavioural, and biological factors likely explain the high prevalence observed in this university cohort.

### 4.2 Gender differences in anaemia prevalence: a critical look at male university students

The prevalence of anaemia among females in our study (60.7%) was substantially higher than the values reported in previous studies conducted both locally and regionally. Reported rates among women of childbearing age range from 16% in Lebanon to 40% in Saudi Arabia and up to 47% in Egypt (7, 51–53). In the UAE, prevalence estimates among female university students have ranged from 18.1 to 26.7% (7, 11, 12). In contrast, our findings reveal a markedly higher rate of 60.70%.

These differences may partly reflect lifestyle and dietary factors. Our study documented insufficient dietary intake among female participants, with many failing to meet nutritional recommendations for key nutrients, including protein, several vitamins, and minerals such as iron. Methodological differences in haemoglobin assessment may also contribute to discrepancies across studies, as mentioned in the previous paragraph. As expected, anaemia was more prevalent among females, consistent with global and regional data indicating a higher risk due to menstruation and reproductive physiology (1, 3, 11, 12). However, the prevalence in males (25.60%) was notably higher than values previously reported in the UAE (2.7%) (19) and in other countries in the region, such as Saudi Arabia, where the prevalence among male university students was 11.1% (21).

In contrast to females, male participants in our study tended to overconsume protein, and more than half reported iron intakes exceeding the recommended dietary allowance. This may be attributable to dietary choices, particularly the regular inclusion of animal-based protein sources. A recent study among UAE adults reported that the majority consume meat, chicken, or fish at least once daily (54). Furthermore, traditional Emirati cuisine emphasises protein-rich staples such as grilled meats from camel, goat, and sheep. The per-capita meat consumption in the UAE has been estimated at 85.1 kg, nearly 18 times the global average, reflecting a strong cultural attachment to meat (54).

Despite these seemingly protective dietary habits, the unexpectedly high prevalence of anaemia among male university students suggests they represent an under-recognised at-risk group. This finding challenges the conventional focus on female populations and highlights the need for more inclusive screening, prevention, and intervention strategies that account for male-specific risk factors.

### 4.3 Association between body composition and anaemia and the obesity paradox among males

In male students, several body composition parameters, including height, fat-free mass, muscle mass, and bone mass, were significantly associated with anaemia, while fat mass showed no relationship. These findings align with research suggesting that lean body mass is positively associated with erythropoiesis and haemoglobin (Hb). Muscle mass was connected to anaemia, especially in men (55). Bone mass was also identified as a factor of anaemia (56). However, in our study, when the body composition parameters were included in multivariable logistic regression, only height remained a significant independent predictor of anaemia.

This relationship likely reflects the cumulative impact of nutritional deprivation during critical growth periods, suggesting that shorter adult stature may serve as a proxy for long-term undernutrition and related micronutrient deficiencies. Given that height is primarily determined by early-life nutrition and is fixed by adulthood, it provides a valuable indicator of earlier-life exposures that influence present health outcomes. Several studies support our findings. For example, Shimizu et al. (56) reported an inverse association between height and anaemia among rural Japanese men, where each standard deviation increase in height was associated with a significantly reduced risk of 41% of the odds of anaemia (OR 0.59, 95% CI 0.45–0.77) in non-drinkers. Although many studies in low- and middle-income countries have focused on children and adolescents, similar trends have been observed, where stunting is consistently associated with anaemia, supporting the use of height as a retrospective marker of cumulative nutritional inadequacy (57, 58). From a public health perspective, these findings reinforce the importance of addressing nutritional inadequacies in early life, especially among males, to mitigate long-term consequences such as anaemia in young adulthood. This highlights the need for integrated growth monitoring and micronutrient intervention programmes from an early age.

Interestingly, despite 55% of male participants being classified as overweight or obese based on BMI, fat mass was not associated with anaemia. Obesity has been shown to disrupt iron homeostasis through inflammation-induced hepcidin upregulation, impaired iron absorption, and altered iron distribution, all of which can contribute to anaemia (59). The absence of an observed association between overweight/obesity and anaemia in our study may reflect the heterogeneity of obesity-related inflammation or the compensatory effects of higher dietary iron intake among this group. As mentioned earlier, most male participants reported iron intakes exceeding the dietary reference values. However, excess adiposity may still reduce iron bioavailability despite sufficient intake, potentially contributing to functional iron deficiency.

A key explanation may lie in the limitations of BMI as an indicator of body composition. BMI does not differentiate between fat and lean mass, and in this population, muscle mass represented a significant proportion of total body weight. As previously discussed, muscle mass and fat-free mass were inversely associated with anaemia, suggesting a protective effect of lean tissue. Therefore, the presence of overweight or obesity in this context may not necessarily indicate excess fat mass but could reflect greater muscle mass in some individuals. These findings imply that in male university students, lean tissue mass and height are likely more clinically relevant predictors of anaemia than adiposity.

### 4.4 Lack of association between nationality and anaemia

Contrary to evidence from regional studies suggesting differences in anaemia prevalence by nationality due to dietary or cultural factors (37, 38), our findings showed no significant association between anaemia and nationality. This may reflect the homogenised lifestyle and dietary patterns among university students, irrespective of cultural background. Alternatively, structural determinants, such as food availability and access within campus environments, may overshadow cultural dietary practises in influencing the risk of anaemia.

### 4.5 Lack of association between dietary intake and anaemia in females

Surprisingly, no significant relationships were found between dietary intake of macro- or micronutrients and anaemia among female students. Potential underreporting in dietary recalls or the influence of non-dietary causes such as menstrual blood loss or recent infection might explain these results (1).

### 4.6 Dietary intake and anaemia in males: role of iron and zinc

In contrast, males with anaemia had lower dietary intakes of iron and zinc. Although these differences were not statistically significant in the multivariate model after adjustment for height and fat-free mass, they are consistent with previous studies, which have shown that both nutrients are essential for making red blood cells and supporting the immune system (60). The results suggest that insufficient intake of iron and zinc may play a role, highlighting the potential value of nutrition-focused strategies in preventing anaemia among male students.

### 4.7 Strengths and limitations

Key strengths of this study include a large sample size with standardised assessments of body composition and haemoglobin levels, as well as sex-stratified analyses. The study also benefits from being the first to comprehensively evaluate anaemia prevalence among male university students in the UAE.

However, some limitations must be acknowledged. The cross-sectional design limits the ability to draw causal inferences. Utilising a point-of-care device to assess anaemia prevalence may have resulted in a slight underestimation of Hb levels. This is a limitation of capillary blood sampling, which can occasionally yield lower accuracy compared to venous blood analysis performed via standard laboratory haematology analyser (50, 58). Reliance on a single 24-h dietary recall may not accurately reflect usual intake, particularly for micronutrients, which are subject to day-to-day variation. Furthermore, the study did not include biochemical markers for iron or other micronutrients such as ferritin, CRP, Vitamin B12 and folate levels, which limits the ability to interpret underlying mechanisms of anaemia. Residual confounding is also possible, as unmeasured or imprecisely measured factors, such as menstrual characteristics, infection history, physical activity, may have influenced the observed associations.

## 5 Conclusion

This study revealed a very high prevalence of anaemia among university students in the UAE, affecting almost half of participants and occurring more frequently in females, while also identifying a substantial proportion of affected males (24.60%), an under-recognised at-risk group. In males, anaemia was associated with body composition, particularly shorter height, and height remained the only independent predictor, suggesting a possible role of early-life nutrition. In females, no clear associations with diet or body composition were observed, indicating that other factors such as menstrual blood loss may be important. Nationality was not associated with anaemia, suggesting shared lifestyle patterns among students. These findings highlight the need for inclusive screening strategies, early nutritional interventions, and targeted health education to reduce anaemia risk among university students in the UAE.

## Data Availability

The raw data supporting the conclusions of this article will be made available by the authors, without undue reservation.
